# Anesthesic Management for Escobar Syndrome: Case Report

**DOI:** 10.1155/2011/515719

**Published:** 2011-03-30

**Authors:** Ayse Hande Arpaci, Fusun Bozkirli, Onur Konuk

**Affiliations:** ^1^Department of Anesthesiology and Reanimation, Faculty of Medicine, Gazi University, 06500 Ankara, Turkey; ^2^Department of Ophthalmology, Faculty of Medicine, Gazi University, 06500 Ankara, Turkey

## Abstract

Escobar syndrome is a rare autosomal recessive disorder which is characterized by growth retardation, axillary, antecubital, popliteal digital, and intercrural joint flexion contracture, pterygium in the eyes, cleft palate, decreased lung capacity, genital abnormalities, and spinal deformity. In this case, we presented the anesthesic management of a 2-year-old child undergoing frontal sling operation for ptosis and amblyopia etiology exploration.

## 1. Introduction

Escobar syndrome is a rare autosomal recessive disorder which is also called “multiple pterygium syndrome” characterized by growth retardation, axillary, antecubital, popliteal digital and intercrural joint flexion contracture, pterygium in the eyes, cleft palate, decreased lung capacity, genital abnormalities, and spinal deformity [1–6]. Intrauterine deaths, congenital respiratory system diseases, short stature, lowset ears, low hairline, arachnodactyly, and cryptorchidism are seen [7]. An operation may be needed in Escobar syndrome for cleft palate, bands between maxilla and mandibula, adhesion of palate to tonsilla, syndactyly, scoliosis, pes equinovarus, genital abnormalities, umbilical or inguinal hernia, and congenital hip dislocation [8].

In this case study, a child diagnosed with Escobar syndrome was presented for anesthesic management undergoing frontal sling operation for ptosis and amblyopia etiology exploration.

## 2. Case

**Figure 1:**
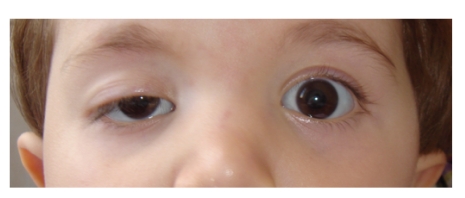
Preoperative photograph of child with ptosis and amblyopia in right eye.

A 2-year-old girl weighing 10.5 kg and 80 cm tall was admitted to an ophthalmology clinic for ptosis (Figure 1). The examination revealed amblyopia, and the child was consulted to anesthesia clinic for preoperative evaluation for frontal sling operation.

The patient had bilateral hypertrophic tonsilla, postnasal mucoid discharge, ptosis in right eye, flexion contracture of right knee, and neck flexion stiffness. Microbial analysis of throat was normal. In posteroanterior chest X-ray cardiothoracic ratio was increased, local eventration of right hemidiaphragm, mediastinal expansion due to enlarged thymus was revealed. The electrocardiography was found normal, and the child was consulted to cardiology department for cardiomegaly. Elective echocardiography was planned, and cardiology reported no additional risk factor for anesthesia. Routine laboratory test results were normal. In preoperative evaluation heart rate was 120 bpm, respiratory rate was 24 breaths/min, and blood pressure was 95/60 mmHg. The patient was assigned as ASA II and scheduled for operation under general anesthesia. 

The patient was brought to the operation theatre without premedication, and heart rate (HR), blood pressure (BP), and peripheral oxygen saturation (SpO_2_) were monitorized. Anesthesia was induced with 8% sevoflurane 100% oxygen mixture, then intravenous cannulation was done. Anesthesia was maintained with 4% sevoflurane 50/50% oxygen-nitrous oxide mixture. Hemodynamic parameters remained stable during deep anesthesia. Airway was maintained by laryngeal mask airway (LMA), number 2, and sevoflurane concentration was reduced to 2% during the rest of the operation. After surgical preparation, local anesthesia was performed. The operation continued for 45 minutes, and no additional anesthetic agent was needed. At the end of the operation anesthesia was stopped, and 100% oxygen was given. Superficial reflexes returned after 3 minutes, and LMA was removed after full wakefulness. The patient was transported to postanesthesia care unit and observed for 30 minutes for vital signs then a transported to a ward without any problems.

## 3. Discussion

Escobar syndrome is a rare disorder characterized by growth retardation, flexion contractures of neck, axilla, antecubital, popliteal, digital and intercrural joints accompanied by multiple pterygia, ptosis, genital abnormalities, and cleft palate [3]. This syndrome is progressive, and 20% lung capacity reduction and spinal deformity are common [4]. 

Escobar syndrome was first described in 3 siblings with ptosis, epicanthal folds, cleft palate, low set ears, retrognathia, downward-turned corners of mouth, syndactily and camptodactyly, talipes equinovarus and rocker bottom feet [3].

The presented case had cervical joint stiffness, ptosis of right eye, and knee flexion contracture. Tonsilla was bilaterally hypertrophic so throat culture was studied and found negative.

When the age of the patient, speciality, and location of the operation were taken into account-general anesthesia was planned. In the literature, a 12-week old neonate was failed to intubate by fiberoptic bronchoscope orally and nasally; then, LMA was placed and through LMA endotracheal tube was inserted by fiberoptic bronchoscope. Also, there is another case of difficult intubation scheduled for spinal instrumentation operation [8]. 

In Escobar syndrome, gamma subunit of acetyl choline receptor which has a role in the muscle-relaxant effect was mutated, and there was inadequate studies investigating other subgroups [7]. So, in the case presented, we resented the use of any muscle relaxant, and anesthesia was deepened by inhalation anesthesia. 

In the case presented, endotracheal intubation was not preferred because of hypertrophic tonsilla and cervical joint stiffness; also, in the literature, cases with acetyl choline receptor subunit abnormalities and cases taken to the intensive care unit because of lung infection, dyspnea, and apnea were reported [5]. We managed airway with LMA under inhalation anesthesia without muscle relaxants.

Airway was secured, and anesthesia was maintained by sevoflurane, and analgesia was provided by nitrous oxide and local anesthetic performed by the surgeon. Although there is a case report in the literature complicated by malignant hyperthermia [9], Kachko et al. [10] presented a case report where general anesthesia and epidural anesthesia was performed for femur osteotomy to a 16-year-old child and stated in Escobar syndrome there is low risk of malignant hyperthermia. There are reported cases where general anesthesia was performed uneventfully [10–12], so we also preferred sevoflurane for anesthesia maintenance. No additional analgesia was required during the operation. The patient was hemodynamically stable during the operation lasting 45 minutes and regained reflexes and recovered from anesthesia shortly after discontinuation of anesthesia. LMA was removed, and the patient was monitored in the postoperative care unit without any incident.

## 4. Conclusion

Genetic syndromes are a challenge for pediatric anesthesia, although this case was managed without any incident. Preoperative evaluation is essential for pediatric patients with multiple abnormalities to accomplish necessary arrangements in advance. We concluded that, in similar cases with probable mutation of acetyl choline receptor and difficult intubation, LMA insertion is a reliable method for security for the anesthetist and the patient.
